# Impacts of high dose 3.5 GHz cellphone radiofrequency on zebrafish embryonic development

**DOI:** 10.1371/journal.pone.0235869

**Published:** 2020-07-09

**Authors:** Subham Dasgupta, Guangxin Wang, Michael T. Simonich, Tingwei Zhang, Lisa Truong, Huaping Liu, Robyn L. Tanguay

**Affiliations:** 1 Department of Environmental and Molecular Toxicology, The Sinnhuber Aquatic Research Laboratory (SARL), Oregon State University, Corvallis, Oregon, United States of America; 2 School of Electrical Engineering and Computer Science, Oregon State University, Corvallis, Oregon, United States of America; Information Technology University, PAKISTAN

## Abstract

The rapid deployment of 5G spectrum by the telecommunication industry is intended to promote better connectivity and data integration among various industries. However, since exposures to radio frequency radiations (RFR) >2.4 GHz are still uncommon, concerns about their potential health impacts are ongoing. In this study, we used the embryonic zebrafish model to assess the impacts of a 3.5 GHz RFR on biology- a frequency typically used by 5G-enabled cell phones and lies within the 4G and 5G bandwidth. We established a plate-based exposure setup for RFRs, exposed developing zebrafish to 3.5 GHz RFR, specific absorption rate (SAR) ≈ 8.27 W/Kg from 6 h post fertilization (hpf) to 48 hpf, and measured a battery of morphological and behavioral endpoints at 120 hpf. Our results revealed no significant impacts on mortality, morphology or photomotor response and a modest inhibition of startle response suggesting some levels of sensorimotor disruptions. This suggests that the cell phone radiations at low GHz-level frequencies are likely benign, with subtle sensorimotor effects. Through this assessment, we have established a robust setup for zebrafish RFR exposures readily amenable to testing various powers and frequencies. Future developmental exposure studies in zebrafish will evaluate a wider portion of the radio frequency spectrum to discover the bioactive regions, the potential molecular targets of RFR and the potential long-term effects on adult behavior.

## Introduction

The advancement of wireless communication technologies over the past decade provides faster connectivity and more bandwidth to a wider population integrating commerce, education, healthcare and consumer applications through interconnected devices. A recent step forward is the 5^th^ generation or 5G wireless technology, currently under a gradual coverage schedule limited by the rate at which necessary infrastructure upgrades can be deployed. The 5G spectrum will cover radio frequencies from < 1 GHz to microwave frequencies up 300 GHz; part of these frequencies (< 5 GHz) overlap with existing 4G LTE and WiFi spectrums. Low band spectrums (<1 GHz) will enable connection to remote areas, whereas mid-band (1–6 GHz) and the spectrum range >24 GHz will ensure better and wider connectivity to individual devices as well as device-to-device connectivity through the Internet of Things (IoT) [1, 2]. As we move to higher spectrum ranges transmitted among our devices, the potential for health effects from higher radiofrequency radiation (RFR) should be examined.

Although no part of the radio or microwave range constitutes ionizing radiation, several studies have associated RFR exposures with adverse health effects such as neuropsychiatric problems, carcinogenicity, neurodegenerative diseases, genotoxicity, lowered sperm quality and impacts on the circulatory, immune, endocrine and skeletal systems [3–6]. Some of these effects are almost certainly thermal in origin, resulting from tissue absorption of higher energy photons with increasing frequency [5, 7]. Thermal effects are readily sensed and easily mitigated, and the ability of RFR to penetrate tissue declines linearly with increasing frequency [8]. Despite many efforts, RFR impacts on biology by non-thermal mechanism(s) remain poorly established, but not yet discountable either.

The impact of RFR on developmental health, in particular, needs better assessment. Developmental stages are the most sensitive to external stressors because the full repertoire of biological targets and molecular processes are operational during organogenesis. Embryonic development thus represents the ideal biological window to determine whether external stressors can interact with and perturb biological functions. Subtle adverse effects on development can also have long term consequences. Recently, the National Toxicology Program conducted a chronic 2-year study on the effects of relatively high dose RFR on carcinogenesis in rats (900 MHz exposure; up to a specific absorption rate (SAR) of 6 W/kg and mice (1900 MHz exposure; up to SAR of 10 W/kg), with RFR exposures beginning *in utero* during the prenatal phase of the test animals. For comparison, the basic limits for human whole-body exposure in terms of SAR are 0.4 W/kg in occupational settings and 0.08 W/kg for general public exposure [9]. The study revealed reduced body weight of both male and female pups and low but significant incidences of malignant gliomas in the brain and schwannomas in the heart of male rats [10]. Follow-up assessments from this study revealed significant DNA damage in the brain and peripheral blood leukocytes of rats and mice [11]. This, and a limited number of other mammal-based studies [5] suggest that developmental exposures to RFR can result in adverse effects. However, there also exists evidence to the contrary, with several epidemiological studies suggesting a lack of effects unequivocally attributable to RFR exposure [12]. Most of the published RFR studies are based on previous generations of telecommunication RFRs. With the advent of 5G RFR exposures from mobile phones and other near proximity devices such as Wi-Fi routers and IoT appliances, public concerns about the safety of these high frequency exposures is likely to increase. This will also lead to a plethora of pseudoscience and well-meaning but poorly informed opinion. Thus, good science in the form of carefully controlled studies aimed at non-thermal effects and mechanisms of higher frequency RFR exposures are necessary and timely.

Since RFR exposures are not chemical in nature, the potential biological mechanisms and targets are expected to be very different from what we are more familiar with, but also readily translatable. For example, chemical receptor mechanisms with species-specific variation in receptor structure or downstream signaling processes may limit the translation of toxicological data across species. Because RFR exposures are electromagnetic radiation, one would expect non-thermal toxicological results, if they exist, to also be operant in humans. In this study, we used zebrafish as a surrogate model to study how RFRs might affect early development. Zebrafish constitutes an ideal model for this study since 1) they undergo rapid development, accomplishing primary organogenesis within 48 h post fertilization, 2) their *ex utero* development greatly facilitates monitoring abnormalities and examination of temporal windows of sensitivity to a stressor 3) RFR exposures of hundreds of embryos can be done uniformly in a multi-well plate inside a small Faraday cage built for contained RFR exposures [13]. Previous studies have assessed the effects of RFR on adult zebrafish. In one study, 2300 MHz 4G RFR (estimated SAR of 0.004 W/Kg) exposure from close proximity to a mobile phone was associated with altered patterns of locomotor activity that were dependent on both time of exposure (morning vs. evening) and duration of exposure, suggesting that RFR affected circadian rhythm [14]. In another study, a 14-day exposure to RFR from a mobile phone emitting ~ 900 MHz (estimated SAR of 0.004 W/Kg) by playing 1 h of music daily via a call from another phone resulted in neurobehavioral and social deficits in adult fish; these effects were also associated with oxidative stress in brain tissues [15]. Overall, these studies suggested that relatively low dose RFR exposures altered neurobehavioral patterns in adult zebrafish. To our knowledge, only one study assessed the effects of developmental RFR exposures in zebrafish where exposure to 100 MHz RFR (SAR ≈ 0.04 W/Kg) from 0 to 72 h post fertilization (hpf) was associated with reduced growth, oxidative stress, increased apoptosis and altered cholesterol pathway [16]. This suggests that vertebrate development may be impacted by RFR and that more research is needed to discover the developmental effects and targets of RFR. The advantages of the zebrafish model should enable rapid study of developmental stage sensitivity to varying RFR frequency and field strength, specifically GHz levels of RFR that define much of the existing and proposed cellular frequency spectrums. To achieve this, we standardized a plate based RFR exposure chamber and conducted a multi-part study assessing potential effects of continuous developmental exposure to high dose 3.5 GHz RFR, SAR ≈ 8.27 W/Kg. We used a transmission range of 60 mm through air and 5 mm through water. The 3.5 GHz frequency has been allocated by the US Federal Communications Commission (FCC) for wireless device manufacturers for the 5G spectrum [17] and is within a frequency range used by 4G and 5G bandwidths. This manuscript reports the first part of this systematic study where we used rapid screening to assess RFR-induced impacts on embryonic development and early life stage behavior.

## Materials and methods

### RFR exposure setup

**Fig 1:**
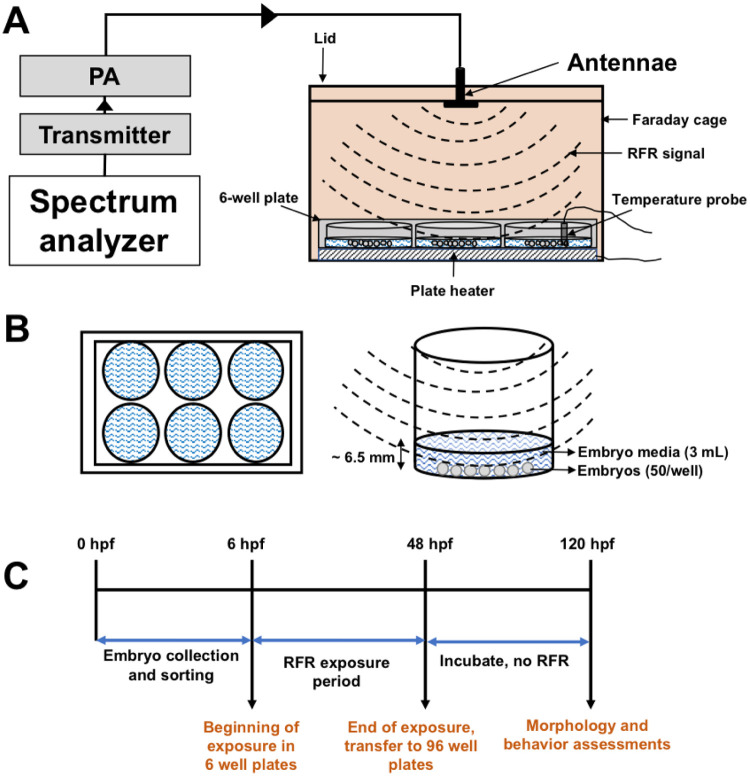
RFR embryonic exposure setup. (A) The transmitter generates RFR signal at 3.5 GHz that is amplified by the PA and broadcast in the Faraday cage via antenna. The Faraday cage contains a 6-well plate on a heating pad that maintains temperature of the plate at ~28°C. Each plate is fitted with a temperature probe to measure temperature of the EM. Arrows represent direction of current. An image of the actual setup is included in S1 Fig. (B) View of the 6-well plate from the top (left) and a magnified view of each well from the side (right). Each well contains ~ 3 mL embryo media and 50 embryos. (C) Experimental flow chart, depicting start of exposure (6 hpf), end of exposure (48 hpf) and time of data acquisition (120 hpf).

The exposure chambers (both for RFR exposure and sham control exposure) consisted of in-house constructed 110 x 80 x 80 mm (L x W x H) Faraday cages made of 20-gauge copper plate with a tight-fitting lid made of the same material. Electrical continuity between the cage and lid was verified with an Ohm meter. The Faraday cages were sized to accommodate a 6 well microtiter plate. The RFR exposure chamber lid was fitted with a Fractus (model FR05-107) ultra-wide band, omnidirectional antenna with a 3.1–5 GHz frequency range, 84% average efficiency, measuring 10 x 10 x 0.8 mm in dimensions. The SMC bulkhead fitting at the antenna passed through the lid of the Faraday cage, insulated from the copper with a nylon sleeve around the SMC barrel and nylon washers behind the upper and lower mounting nuts. The Fractus antenna was thus centrally affixed 3 mm below the inside surface of the Faraday cage lid and was immobile. The sham control chamber was identical to the RFR exposure chamber, but without an antenna and served to shield against the impacts of background RFR present at the location of the experiment. Fig 1A and 1B show our experimental setup for RFR exposure where the signal was generated using a transmitter (Analog Devices, model ADRV9364-Z7020) connected to a power amplifier (PA) (Mini-circuits ZHL-42+). Using a spectrum analyzer (Tektronix RSA3408A) with a 30 dBm measurement threshold, we performed preliminary assessments to measure the output from the PA. With a transmitter output of -9 dBm at 3.5 GHz, the PA output was 26.7 dBm. During our experiments, we set the transmitter to an output of -6 dBm at 3.5 GHz and expected a PA output of 30–32 dBm signal power. The amplified signal was administered to the zebrafish embryos via SMC cable connection from the PA to antenna attached to the RFR chamber; the sham chamber did not receive any signal.

The incident power density of the zebrafish embryo exposures, at the water surface, was approximately 22 W/m^2^. This estimate considered the reported signal power, a 6 cm distance (≈0.2λ) from the antenna and unity gain at the antenna for use in the equation:

$$P_{D}=\frac{P_{o}G_{tx}}{4\pi D^{2}}$$

Where: P_D_ = W/m^2^; P_o_ = output W from antenna; G_tx_ = antenna gain; D = distance from the antenna in meters. Because of the high complexity in calculating the propagation path loss in near-field propagation cases such as this, we believe that in our small volume, highly reflective system, using the density equation provides sufficient insight.

To report the RFR exposures in more widely accepted health safety terms, we estimated the specific absorption rate (SAR) to be 8.27 W/Kg. SAR is defined as the rate at which RF energy is absorbed per unit mass, i.e., the ‘dose rate’ in watts per kilogram (W/kg) using the equation:

$$SAR=\frac{\sigma E^{2}}{m_{d}}$$

Where σ = conductivity of material (here zebrafish tissue, assumed isotonic with surrounding embryo medium which is approximately 1 Siemen /m conductivity); E = electric field in V/m, where the incident power density of 22 W/m^2^ (above) is equal to 91 V/m; m_d_ = the mass density of embryonic zebrafish, estimated from wet embryo weight, at approximately 1 mg/mm^3^ or 1000 Kg/m^3^.

Each chamber accommodated a Cell MicroControls well plate heater (model HWPT-96) controlled remotely by an mTCII micro-temperature controller to maintain a constant ~ 28°C during embryonic development. The wire leads for the plate heater and thermistor probe passed through a small hole drilled on the short edge of the cage bottom just above the cage floor. The stack height (bottom to top) of cardboard thermal insulation, plate heater and the well plate was 25 mm. For the RFR exposure chamber, the distance from the antenna face to the surface of the water column surface was consistently set at 60 mm. We note that 3.5 GHz permittivity in a vacuum (dielectric constant = 1 = perfect) vs. air are so similar that they are equal, for all practical purposes, over a 60 mm transmission range (the higher the dielectric constant of a substance, the greater the signal attenuation). To reduce signal attenuation by the water component (1000 μS/m conductivity, 28°C, thus dielectric constant > 88 and attenuation at least 88 times that of air), the water depth above the embryos was limited to ~5 mm, the minimum required to avoid potentially confounding effects of evaporation. S1 Fig contains an image of the actual experimental setup.

### Zebrafish husbandry

Adult Tropical 5D zebrafish were raised at Sinnhuber Aquatic Research Laboratory (SARL) at Oregon State University. The zebrafish were raised in standard laboratory conditions (28°C with 14 h light: 10 h dark photo cycle). Adult zebrafish were fed size appropriate Gemma Micro (Skretting Inc, Tooele, France) twice daily without supplementation of any live feed [18]. Adult care and reproductive techniques followed approved Institutional Animal Care and Use Committee protocol 5113 at Oregon State University.

### Exposure regime

Embryos were collected in water from our recirculating system and sorted according to Kimmel et al 1995 [19]. All exposures and analyses were done based on our well- established high throughput pipeline (described in [20–23]), with some modifications. At ~6 hpf, embryos were bleached and transferred to 6 well flat-bottom plates (Falcon, Corning) with 50 embryos per well and 1 plate (6 wells) per treatment. The use of multiple embryos per well in a 6 well plate enabled the minimization of spatial variability of RFR exposure across different embryos that may be a concern for 96 well plates with 1 embryo in each well typically used in our pipeline. Furthermore, plastic boundaries within each well of 96 well plates may also attenuate RFRs and distribute uneven signal into each well. All embryos were incubated in embryo media (EM), consisting of 15 mM NaCl, 0.5 mM KCl, 1 mM MgSO_4_, 0.15 mM KH_2_PO_4_, 0.05 mM Na_2_HPO_4_ and 0.7 mM NaHCO_3_ [24]. The volume of EM in each well was minimized at 3 mL to reduce RFR signal attenuation through the 5 mm water column above the embryos; preliminary observations showed that 50 embryos within this volume developed normally. To avoid evaporation, the plates were sealed with ThermaSeal RTS (polyolefin, 50 μm thick) pressure-sensitive film and transferred to the temperature-controlled Faraday cages. A thermocouple temperature probe (Fluke, type K), remotely connected to a handheld thermometer (Fluke model 51–2), was placed into the water column of one well of each plate to serve as a secondary monitor of temperature independent of the thermistor probe attached to each well plate heater. RFR exposure was static, continued from 6–48 hpf and PA output were verified by spectrum analyzer. At 48 hpf, a subset of exposed embryos (N = 48, 8 embryos from each well of the 6 well plate to maintain representation of every well) were transferred into wells of a 96 well plate (1 embryo/well; prefilled with 100 μL) for downstream assessments; no mortality or morphological defects were seen in embryos at 48 hpf. The 96 well plates were then sealed and incubated at 28°C in the dark for until 120 hpf when the plates were run through a battery of morphological and behavioral assessments (see below). The experiments were run thrice to capture the variability and to measure reproducibility; temperature and PA signal output remained stable and consistent throughout the experiments. Fig 1C shows a flow chart representing our experimental paradigm.

### Developmental toxicity assessments

At 120 hpf, mortality, morphology and behavioral endpoints were rapidly assessed; data for each treatment were combined from the 3 separate experiments (N = 48 per experiment) resulting in N = 144. Seventeen developmental morphology endpoints included yolk sac edema (YSE) and pericardial edema (PE); body axis (AXIS), trunk length (TRUN), caudal fin (CFIN), pectoral fin (PFIN), pigmentation (PIG), and somite (SOMI) deformities; eye (EYE), snout (SNOU), jaw (JAW), and otolith (OTIC) malformations; gross brain development (BRAIN); notochord (NC) and circulatory (CIRC) deformities; swim bladder presence and inflation (SWIM); and touch-responses (TR). The presence or absence of abnormality in each endpoint was entered into a laboratory information management system called the Zebrafish Acquisition and Analysis Program (ZAAP) [20]. Behavioral assessments consisted of the larval photomotor response assay and the larval startle response assay using the Viewpoint Behavior Technology ZebraBox and ZebraLab motion tracking (Viewpoint Life Sciences, Lyon, France) and stimulus triggering software; these were conducted just before morphological evaluation. For photomotor response, larvae experience a total of 3 light cycles, each cycle consisting of 3 min of alternating light and dark. The startle response assay consisted of an audible 100 dB, 600 Hz tone occurring for 900 ms, 30 seconds after the conclusion of the photomotor response assay (same instrument platform) with motion tracking commencing at the tone and for the following 9 seconds. Raw data for each assessment is included in S1 File.

### Statistical analyses

All statistical estimations were done within ZAAP which uses the R platform (https://www.r-project.org/) for analyses of various morphological and behavioral endpoints; details of analyses are described in our previous publications [21–23]. For mortality and morphology, statistical significance based on binary responses was computed as described in [21, 22]. Briefly, significant differences between control and exposed fish were computed using a one-sided Fisher’s exact test, where adverse endpoints were tested to have a greater occurrence in exposed fish. For the photomotor response assay, an entropy score was calculated for each light phase interval and compared with the control group to compute a relative ratio, as described in [23]. For the startle response assay, the area under the curve (AUC) and peak response was calculated for the first startle only and compared to the first control startle response. This assay is performed in visible light. For both assays, statistical significance was determined using a Kolmogorov–Smirnov test (p <0.05). For all behavioral assays, dead or severely deformed embryos were excluded from the analyses.

## Results and discussion

**Fig 2:**
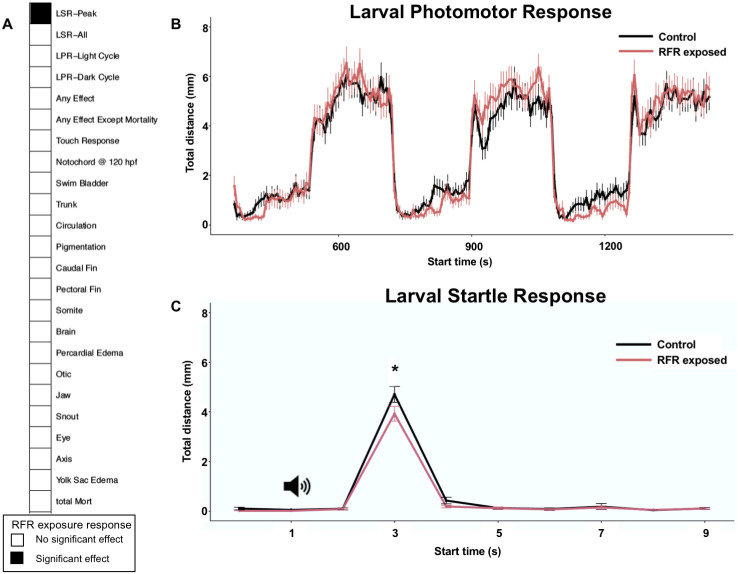
Effects of RFR on embryonic development. Embryos were exposed to 3.5 GHz, ~ 30 dBm RFR, at a specific absorption rate (SAR) ≈ 8.27 W/Kg from 6–48 hpf and developmental parameters were measured at 120 hpf. All assays (panels A-C) were sequentially conducted from the same subset of embryos, with a total of 144 embryos from 3 replicate experiments used per treatment condition. (A) Summary of effects observed within our study. Measured parameters include mortality (Mort), 17 morphological parameters and 2 behavioral parameters (larval photomotor (LPR) and startle (LSR) responses). “Any effect” indicates a combination of all 17 morphological parameters. “LSR-All” indicates AUC and Peak measurements combined. (B) RFR exposure does not have any significant effect on photomotor response. (C) RFR exposure results in reduced peak height for startle response. 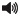 denotes the acoustic signal. * indicates a statistically significant difference at p<0.05.

The primary objective of this study was to assess whether exposure to high dose rate GHz frequency RFR is associated with any developmental perturbations during embryogenesis; this frequency range is expected to be widely deployed by major cellular services and hence most relevant for human exposures [17]. Importantly, we have created a microtiter plate-based format for studying the impacts of RFR that can effectively replicate RFR exposures at different frequencies, field strengths and developmental windows. During the experiment (6–48 hpf), the temperature of the water medium for both controls and RFR-exposed fish remained at ~28–29°C (S1 File), suggesting that, consistent with previous studies [16, 25], RFR exposures did not detectably increase water temperatures in the wells. Additionally, because of their small 1.5–2 mm size and composition, there is no reason to expect that zebrafish embryos would experience any net thermal flux beyond equilibrium with their environment. At 120 hpf, the RFR exposure did not lead to changes in mortality rate or incidences of abnormal morphology (Fig 2A). These results are in contrast to a previous study that showed zebrafish embryonic exposure to 100 MHz RFR, estimated SAR ≈ 0.04 W/Kg, from 0–48 hpf resulted in developmental delays [16]. Disparate results between the two studies may be due to the time of exposure initiation (0 hpf in their study vs. 6 hpf in ours), since early developmental stages (< 6 hpf) span early developmental events (cleavage, blastulation, gastrulation) that can be more sensitive to some stressors. Alternatively, because radio and micro-wave RFR penetration of tissues declines exponentially as frequency increases [8, 26], the 100 MHz exposure may have been substantially more bioactive than our 3.5 GHz exposure. The RFR exposures did not alter the photomotor response, in either the light or the dark phases (Fig 2A and 2B). In the startle response assay, while the areas under the curve between control and RFR exposed groups were not significantly different, the RFR exposure did modestly reduce the peak height (peak swim distance); the acoustic startle was ~16.5% lower in the RFR exposed groups compared to the controls (p = 0.045) (Fig 2A and 2C). The startle response is primarily driven by a sensorimotor response to acoustic cues and has been widely used to detect learning deficiencies in humans and to screen for neuroactive drugs in zebrafish [27, 28]. Suppression of startle response is indicative of depressed sensorimotor function which may result in adverse effects on neurobehavior during post-developmental stages.

Overall, our results did not reveal any large-scale effects of RFR exposure on embryonic survival or development but did reveal a modest depression of sensorimotor function. The possibility remains that developmental RFR exposures may produce molecular or later life stage effects not evaluated in this initial study. For example, the subtle startle response effect could be an early indicator of adult neuropsychiatric outcomes, like those detected in previous RFR studies [6]; though we note that published data suggesting non-thermal RFR effects associated with the low Hz (extreme low frequency; ELF) range is generally more conclusive than the published data suggesting non-thermal effects from exposure to the high MHz—low GHz range. It is also possible that 3.5 GHz RFR at a SAR of 8.27 W/Kg simply does not interact with and perturb normal vertebrate development. The combination of strong signal attenuation by the thin layer of EM (dielectric constant >88) over the embryos may have resulted in incident radiation density below the threshold for significant biological effects [25]. It is to be noted that we employed artificially high signal strength to maximize the likelihood of detecting RFR impacts; the 8.27 W/Kg SAR is approximately 200x larger than what a person using a mobile phone operating at mid-band 5G would be exposed to, and at least 100x larger than the basic limit for whole body exposure of 0.08 W/kg for general public exposure [9]. RFR tissue penetration is predicted from extensive modeling to be no more than about 6 mm at 3.5 GHz and rapidly declining to less than 1 mm in the upper 5G band and above [26]. The lack of large-scale effects at this high signal strength shows that a 3.5 GHz RFR is likely benign to users of devices usually emitting much lower signal strengths. Finally, it is possible that any RFR effects induced in our study may have been transient and the embryos may have recovered from or adapted to the static signal between the end of exposure (48 hpf) and experimental measurements (120 hpf). Future studies should employ longer continuous embryonic exposure periods with rapidly modulated frequencies to mimic mobile network signals. Our exposure platform also provides us an ideal setup for testing higher frequency ranges, including the 5G-specific millimeter-wave range, in the future. In addition, we will also be able to study the compounding effects of RFR and other radiation types or chemical stressors on embryonic physiology.

## Conclusion

Our study suggests that RFR, within the low GHz frequencies, is predominantly benign during embryonic development, but it may mildly depress sensorimotor functions when administered at a dose rate significantly higher than the general public exposure. This is the spectrum portion currently used for 4G LTE and 5G mid-band signals. Importantly, with this established, robust, *in vivo* testing platform, we are ideally positioned to model changing RFR exposures scenarios and measure their biological effects to address the concerns regarding broadband technology and human health.

## Supporting information

S1 Fig Experimental setup for RFR exposure. (TIFF)

S1 File Temperature and raw data for morphological and behavioral assessments following RFR exposures. (XLSX)
