# Supplementary figures and images for: Impacts of high dose 3.5 GHz cellphone radiofrequency on zebrafish embryonic development
Source: PLoS One. 2020 Jul 9;15(7):e0235869. doi: 10.1371/journal.pone.0235869 (PMC7347199; doi:10.1371/journal.pone.0235869)

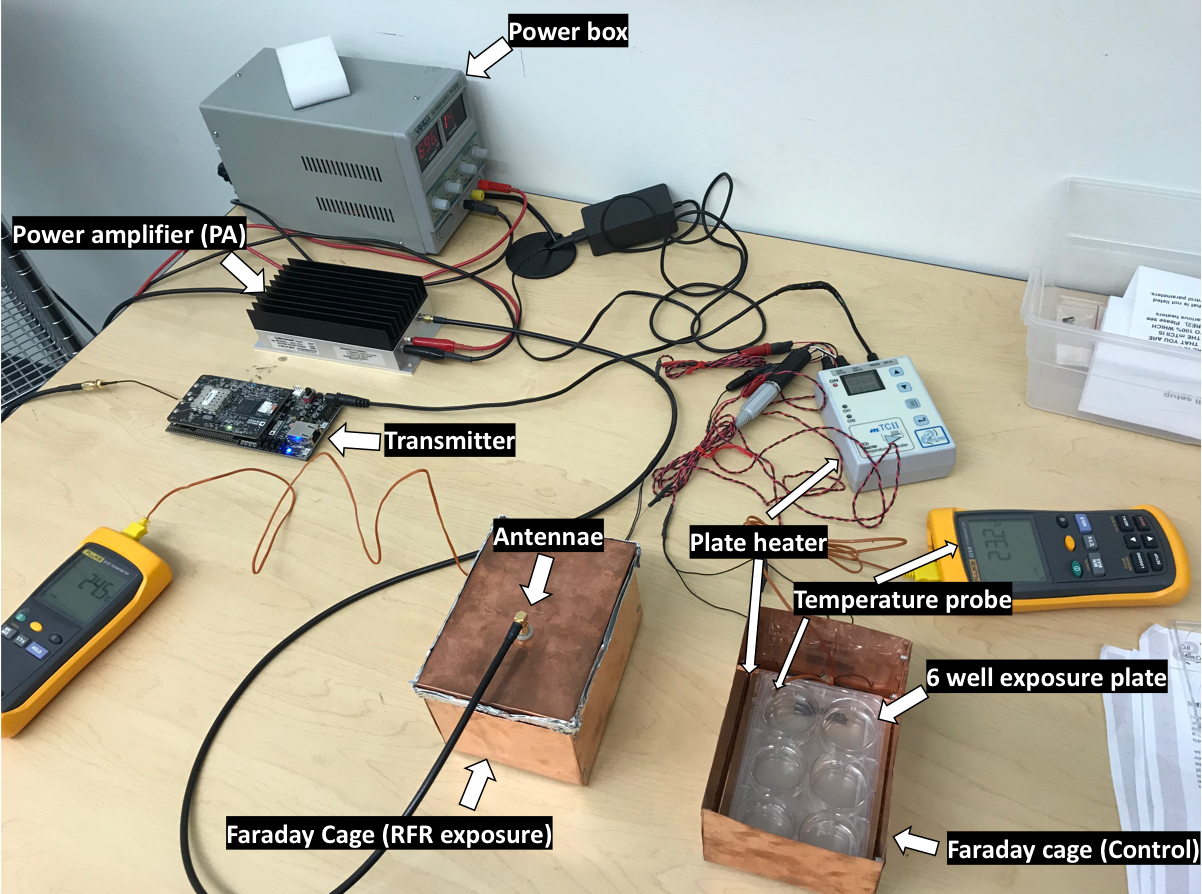

Supplement: S1 Fig — (TIFF) [file pone.0235869.s001.tiff]
